# Inhibition of bacterial toxin recognition of membrane components as an anti-virulence strategy

**DOI:** 10.1186/s13036-018-0138-z

**Published:** 2019-02-19

**Authors:** Eric Krueger, Angela C. Brown

**Affiliations:** Department of Chemical and Biomolecular Engineering, Lehigh University, Bethlehem, PA 18015 USA

**Keywords:** Bacterial toxin, Anti-virulence, Cell membrane, Receptor decoys, Antibiotic resistance

## Abstract

Over recent years, the development of new antibiotics has not kept pace with the rate at which bacteria develop resistance to these drugs. For this reason, many research groups have begun to design and study alternative therapeutics, including molecules to specifically inhibit the virulence of pathogenic bacteria. Because many of these pathogenic bacteria release protein toxins, which cause or exacerbate disease, inhibition of the activity of bacterial toxins is a promising anti-virulence strategy. In this review, we describe several approaches to inhibit the initial interactions of bacterial toxins with host cell membrane components. The mechanisms by which toxins interact with the host cell membrane components have been well-studied over the years, leading to the identification of therapeutic targets, which have been exploited in the work described here. We review efforts to inhibit binding to protein receptors and essential membrane lipid components, complex assembly, and pore formation. Although none of these molecules have yet been demonstrated in clinical trials, the in vitro and in vivo results presented here demonstrate their promise as novel alternatives and/or complements to traditional antibiotics.

## Background

Worldwide, infectious diseases are responsible for 15 million deaths annually, and in lower-income countries, these infections account for almost 60% of deaths [1]. Advances in sanitation and nutrition, as well as the development of vaccines and antibiotics have greatly reduced this statistic over the past century. However, the effectiveness of antibiotics has been reduced in recent years due to increased incidents of resistance in disease-causing organisms.

This troubling situation stems from a number of factors. The overuse and misuse of antibiotics in recent years has led to the rapid selection of antibiotic resistant bacteria and the passing of those resistance genes to other populations. The development of new antibiotics can be difficult, as it requires the identification of a molecule that can specifically target bacterial cells without affecting eukaryotic cells. This difficulty, in addition to the limited profit derived from antibiotics, has led to decreased interest in the field by pharmaceutical companies. Additionally, in the United States, the aging population means that more patients are becoming immunocompromised through disease, chemotherapy, or organ transplantation and/or entering healthcare environments where resistant organisms are prevalent [2]. We are now in the midst of a perfect storm – with an increased number of resistant organisms, a population more susceptible to those organisms, and few effective approaches to treat them.

The development of new antibiotics has traditionally been accomplished by chemically modifying the structures of currently used antibiotics to avoid resistance mechanisms and increase the activity; cephalosporins and carbapenems, for example, are derived from the basic structure of penicillin [3]. Another approach is to combine two drugs with complementary targets, such as Augmentin®, which combines amoxicillin, a drug that is well-tolerated, with clavulanate, which inhibits the β-lactamase enzyme that leads to the development of resistance against amoxicillin [3]. The last completely new class of antibiotics was developed in the 1980’s, and as a result, the current pipeline is seriously lacking in promising drugs [2, 4]; only seven new antibiotic applications were approved by the United States Food and Drug Administration in the years 2000 to 2009, compared to over 30 in the 1980’s [5].

An effective antibiotic must target some aspect of the bacteria that is different from that of the host to properly inhibit bacterial growth without affecting the health of the patient. Three processes that have been found to be distinct enough from their eukaryotic equivalent to be effectively used as targets include: (1) synthesis of the cell wall, (2) synthesis of proteins, and (3) replication/repair of DNA [6]. In addition to targeting a process that is distinct from the eukaryotic equivalent, each of these targeted processes is essential for bacterial survival. While this approach is effective in killing most of the bacteria, it actually facilitates the development of resistance genes through a process known as “selective pressure.” Those bacteria that have some mutation that allows them to resist the applied antibiotic survive, while those that do not have the mutation are killed. The next generation arises from the living bacteria, many of which have the adaptation that allows them to resist the antibiotic. Thus, the antibiotic resistance trait is quickly spread to subsequent generations. Because bacteria grow quickly and are present in high numbers, resistance to new antibiotics inevitably occurs very quickly. In recent years, resistance to new antibiotics has been reported within one-to-two years of the drug’s introduction [5].

In an effort to identify an entirely different approach to the treatment of bacteria, a number of researchers have begun to focus on virulence factors, specific molecules produced by pathogenic bacteria, which allow them to survive within the host. These virulence factors include adhesins that allow the organism to bind to surfaces it would otherwise be unable to bind to, toxins to modulate the host immune response, and enzymes to extract essential nutrients from the host, among many others [7–9]. Most pathogenic organisms produce multiple virulence factors; together, this arsenal allows the pathogenic organism to establish a comfortable niche within the host organism. For example, the primary virulence factors of enterotoxigenic *Escherichia coli* (ETEC) include both surface-associated colonization factors (CFs) [10] and secreted adhesins (TcpA) [11] to enable bacterial colonization in the intestine, along with several secreted toxins, including a heat-labile toxin (LT) and a heat-stable toxin (ST). The toxins induce a cascade that leads to the massive release of water and ions from the targeted cells, which results in the severe diarrhea associated with infection and provides the bacteria a means to spread to other hosts [12].

An antibiotic approach that focuses on inhibiting the virulence of the bacteria would eliminate the advantage provided by the specific virulence factors and promote clearance by the immune system, without facilitating the development of resistance [13]. One side benefit of this approach is that it would specifically target the pathogenic bacteria, leaving the great number of beneficial bacteria in the microbiome intact, thus limiting the unpleasant side-effects of current antibiotics [14]. Additionally, as many of these virulence factors are located outside of the bacterial cell, either on the surface or secreted into the extracellular environment, issues of getting drugs into Gram-negative bacteria, which contain two membranes, are not a concern.

Secreted exotoxins are one class of virulence factor that have been successfully targeted for anti-virulence strategies. These protein toxins are produced by many bacteria, both Gram-positive and Gram-negative, as a means to enhance their colonization in the host. Toxins usually play a significant role in the pathophysiology of disease, and in some cases, such as in ETEC, act specifically as the disease-causing component, making disruption of these pathways an ideal anti-virulence strategy. These toxins interact either within the cytoplasm or on the surface of the host cells to induce a signaling cascade that ultimately leads to cell death. Additionally, some toxins act as cytolysins, permeabilizing the host cell membrane to disrupt the protective barrier of the cell. In all cases, the toxin must first interact with some component of the host cell membrane in order to initiate its toxic mechanism. In this review, we will highlight recent approaches to prevent the activity of a wide array of bacterial toxins by interrupting some aspect of their membrane interaction.

## Fundamental mechanisms of specific bacterial toxin activity

Disruption of toxin activity as an anti-virulence strategy requires an understanding of the key steps in the mechanism by which the toxin interacts with the host cell. With this mechanistic data, targeted molecules can be designed to interfere with specific steps in the pathway. Here, we describe the structure and mechanisms of the toxins to be described in this work, as well as the role of each in the particular disease the toxin and bacteria are associated with.

### *Vibrio cholerae* cholera toxin (CT)

Although relatively rare in industrialized nations, cholera remains a major threat to public health worldwide with over 130,000 cases reported from 38 countries in 2016 [15]. The illness is caused by cholera toxin (CT), one of the predominant virulence factors of the bacterium *Vibrio cholerae* [16, 17]. CT is a member of the AB toxin family and is composed of a single A subunit associated with five B subunits arranged in a pentameric ring [18, 19]. The entire hetero-hexamer complex is assembled in the bacterial periplasm before secretion, where it is subsequently internalized into the host cells through the initial interaction of the B pentamer with the ganglioside GM1 on the cell surface [20]. Only after internalization is the catalytic A subunit activated, which ultimately results in the efflux of ions and water from the cell, causing the severe diarrhea associated with the disease that releases the organism back into the environment [21–25]. The affinity of the CT B pentamer for GM1 initiates the toxic activity of CT, making this specific interaction a focus for receptor-based methods of inhibition.

### *Aggregatibacter actinomycetemcomitans* leukotoxin (LtxA)

*Aggregatibacter actinomycetemcomitans* is associated with aggressive forms of periodontitis [26, 27] as well as systemic infections including endocarditis [28]. Because strains of *A. actinomycetemcomitans* most closely associated with disease have been demonstrated to secrete the most LtxA [29–31], this toxin has been described as a “key” virulence factor of the organism [32]. This immunosuppressive protein specifically targets human white blood cells [33] through its recognition of both cholesterol [34, 35] and the lymphocyte function-associated antigen-1 (LFA-1) integrin [36–39]. Studies have revealed that the interaction of the toxin with the CD11a subunit of LFA-1 is required for toxin activity and is suspected to drive its species specificity [37, 38]. Following binding, the toxin is internalized by endocytosis where it triggers apoptosis through a lysosomal mediated pathway [36, 37]. Unlike other toxins such as CT, the exact cytotoxic pathway of LtxA is not fully understood. Consequently, there are no treatment options to inhibit LtxA activity once inside the host cells, making receptor-based inhibitors the only current method to hinder LtxA activity.

### *Bacillus anthracis* anthrax toxin

*Bacillus anthracis* is a soil-dwelling, spore-forming organism that can cause infections in grazing animals, such as sheep [40]. Humans are less likely to contract disease naturally, as a significant number of spores is required to initiate illness [41]. The bacterium generally resides in a dormant endospore state, where it is largely protected from extreme physical environmental stresses such as heat, desiccation, oxidation, and UV and γ-irradiation [42]. Upon infection of a host, the spores are taken up by macrophages where the favorable environmental conditions trigger their transition to vegetative cells. The cells are released from the macrophages and rapidly multiply in the host’s blood and lymphatic system. In the vegetative state, the cells produce two virulence factors that are proposed to help elude the host’s defense mechanisms: a protective polypeptide capsule and the components of the binary toxin complex [43]. These factors allow the organism to proliferate expeditiously until, acutely overwhelmed by the pathogenic load, the host dies and returns the spores back into the environment [43, 44].

The primary human health concern with this organism and particularly its toxins is the potential nefarious use as a biological warfare agent [45], and thus significant efforts have been made to identify anti-anthrax toxin strategies. The anthrax toxin consists of three components, the protective antigen (PA), which recognizes a host receptor; the lethal factor (LF), which is a metalloprotease; and the edema factor (EF), an adenylate cyclase [46]. In the initial step of cellular intoxication, the PA recognizes either the anthrax toxin receptor (ATR), which is also called tumor endothelial marker 8 (TEM8) [47], or the capillary morphogenesis protein 2 (CMG2) [48]. After binding to its receptor, PA oligomerizes, which facilitates EF and/or LF binding directly to the PA heptamer [46].

### *Staphylococcus aureus* toxins

Despite being a remarkably common bacteria, colonizing the nasopharynx and skin of approximately one-third of the population, *Staphylococcus aureus* is major human pathogen [49]. Infections from *S. aureus* can be extremely dangerous, as strains have become resistant to certain beta-lactam antibiotics, such as methicillin, and contribute to over 11,000 deaths annually [50]. Furthermore, the organism is increasingly becoming resistant to vancomycin, the most common antibiotic used to treat *S. aureus* infections, severely limiting treatment options [50]. As a mechanism to hinder an immune response following infection, *S. aureus* can produce five different pore-forming bicomponent leukocidins that target phagocytes in the host: Panton-Valentine leukocidin (LukSF), leukocidin AB (LukAB), leukocidin ED (LukED) and two γ-hemolysins (HlgAB and HlgCB) [51]. Each leukocidin pore complex is a hetero-oligomer comprised from two types of subunits (S-class and F-class) that assemble after binding to the receptor on the host cell membrane [52, 53]. Once an S-class subunit initially binds to its target, other S- and F-class subunits are recruited and oligomerize, creating an alternating octameric pre-pore structure. The pre-stem structural domains then unfold, penetrating the cell membrane to form a mushroom-shaped β-barrel transmembrane pore. The resulting 2.5 nm diameter channel kills the host cell by osmotic lysis [52].

In addition to the leukocidins, most strains of *S. aureus* produce α-hemolysin, a toxin that is active against many different cell types [54]. The toxin is secreted as a monomer that oligomerizes after insertion in the membrane to form heptameric channels, which cause cell lysis [55]. At low concentrations, the binding of the monomers to the cell surface seems to be driven by an unidentified protein receptor, but at high concentrations, the toxin interacts nonspecifically with the membrane lipids [56].

### *Clostridium perfringens* ε-toxin

*Clostridium perfringens* is a group of Gram-negative spore-forming anaerobic bacteria responsible for disease in both humans and food production animals [57]. Diseases associated with *C. perfringens* infections are typically toxin-mediated [57]. Remarkably, there are five strains of *C. perfringens* (A–E), which combined, produce more than 15 different toxins [58]. The specific function of each of these toxins is not known, but, as in other spore-forming bacteria, they are likely critical to the successful reproduction of the organism after infection of the host. Of these toxins, the epsilon- (ε-) toxin is by far the most toxic *C. perfringens* toxin and one of the most lethal bacterial toxins behind only the neurotoxins produced by *Clostridium botulinum* and *Clostridium tetani* [58, 59]. The ε-toxin is produced by *C. perfringens* types B and D and is most frequently found to infect sheep and goats but has also been known to affect humans [59]. Currently, there are no vaccines or treatments against ε-toxin approved for human use, and due to its potency, this toxin is considered a Category B bioterrorism agent by the Centers for Disease Control and Prevention [60]. The ε-toxin is a pore-forming toxin comprised of three domains responsible for receptor binding, membrane insertion for channel formation and proteolytic activation [58]. The monomeric toxin in solution is activated by cleavage of the proteolysis domain and subsequently interacts with caveolin-1 and -2 in lipid rafts to form a heptameric pre-pore on the cell surface [61]. After a conformational change, the membrane insertion domain penetrates the plasma membrane, forming a 2 nm diameter pore, which disrupts the ion gradients and membrane potential of the cell, leading to cell death [58].

### *Helicobacter pylori* vacuolating toxin (VacA)

One of the few bacteria directly linked to cancer, *Helicobacter pylori* is a Gram-negative bacterium colonizing the gastric mucosa of humans and is one of the most common bacterial infections worldwide [62, 63]. *H. pylori* has been classified by the World Health Organization as a group 1 carcinogen as a major risk factor for gastric cancer, being considered analogous to smoking and lung cancer [64, 65]. The pathogen produces several virulence factors that may aid in its survival in the unique niche of the stomach. The secretion of urease buffers the local pH before the bacteria enter the mucus layer of the stomach, and a neutrophil activating protein (HPNAP) enables the release of nutrients from the mucosa to promote colonization [66]. One of the key virulence factors of *H. pylori* is the vacuolating toxin, VacA, which, as its name suggests, induces cytoplasmic vacuoles in cultured host cells. VacA has been proposed to support bacterial survival by increasing the permeability of the host cell membranes, thus supplying the organism with nutrients [67]. However, a comprehensive mechanism of the VacA cytotoxic pathway remains unclear [68]. In solution, the toxin forms oligomeric complexes, but upon extracellular acidification, the complexes disperse, bind to host cells and reassemble to form anion-selective channels in the plasma membrane. In addition to the multiple proteinaceous cell membrane receptors for VacA that have been proposed, sphingomyelin has been reported to be essential for toxin functionality, suggesting a role for lipid rafts in the toxic activity [69]. After binding, VacA internalization has been shown to rely on GPI-anchored proteins in a clathrin-independent pinocytosis pathway [70–72]. Curiously, unlike many other internalized bacterial toxins, VacA does not have any known enzymatic activity [73]. Instead, VacA is ultimately trafficked to the mitochondrial membrane, where it disrupts morphological dynamics, resulting in apoptosis [74]. Although many studies have been conducted on the VacA toxin, the evidence has revealed a wide spectrum of mechanistic pathways that continue to necessitate more investigation into its cytotoxic activity.

### *Streptococcus pneumoniae* pneumolysin (Ply)

Infections from *Streptococcus pneumoniae* are a significant cause of morbidity and death, resulting in an estimated 1.6 million deaths worldwide, including approximately 0.7–1 million children under 5 years of age [75]. While vaccination remains one of the most important preventative measures, neither of the current vaccines, the capsular polysaccharide or the protein-polysaccharide conjugate, offer a wide serotype coverage [76]. However, one of its predominant virulence factors, pneumolysin (Ply), is highly conserved among *S. pneumoniae* strains, making it a good candidate for therapeutic development [77]. Ply is a cholesterol-dependent cytolysin (CDC), forming ring-shaped pores in cholesterol-containing membranes. After binding to cholesterol in a host cell membrane, Ply oligomerizes into a 30–50 subunit pre-pore complex before inserting into the membrane forming a 26 nm diameter channel [78]. By disrupting membrane integrity, Ply releases nutrients from the host cells to further facilitate *S. pneumoniae* colonization. It has also been shown to target ciliated bronchial epithelial cells, disrupting the function and integrity of the bronchial epithelial layer, which may impede the clearing of mucus from the lower respiratory tract and/or permit the pathogen to enter the bloodstream [79, 80]. A recent study hypothesized that Ply-induced inflammation may contribute to pathogen transmission. In mouse models, Zafar and colleagues determined that Ply-stimulated inflammation of the upper respiratory tract resulted in increased bacterial shedding and nasal secretions, suggesting that the toxin plays a role in transmitting *S. pneumoniae* to other hosts [81].

### *Clostridium difficile* toxins

The bacterium *Clostridium difficile* is one of the leading causes of hospital-associated illnesses, which ironically, often arises because of antibiotic treatment for an unrelated infection [82, 83]. Like other *Clostridium* species, *C. difficile* is a spore-forming bacterium, enabling it to withstand extreme conditions like those used to disinfect hospital facilities. The endospore state is essential for infection and transmission of the organism, protecting it as it moves through the stomach and into the small intestine during infection and after being shed into the environment in host feces [84]. Upon transitioning into the active vegetative state, the pathogen begins producing toxins that are responsible for disease. Its two major virulence factors, TcdA and TcdB, are large multi-domain toxins that enter the host cells through endocytosis after binding to cell surface receptors. While both toxins share similar mechanisms of cytotoxic activity, TcdB has a higher potency and has been the recent focus for inhibition. The 270 kDa TcdB toxin consists of the enzymatic N-terminus region, subdomain A, and the C-terminal subdomain B, responsible for receptor binding and pore formation [85, 86]. Within subdomain B is the combined repetitive oligopeptides (CROPs) region, which is considered to be the receptor binding domain [87]. After binding, the toxin is internalized by endocytosis where it undergoes conformational changes due to the acidic endosomal pH, exposing the pore forming domain, which then translocates across the endosomal membrane. The catalytic subdomain A then passes through the pore where it is cleaved and released into the cytosol to implement its cytotoxic activity [85, 88]. The exact role of the TcdA and TcdB toxins in disease remains unclear [84, 85]. As the bacterial colony moves into the stationary phase of growth, there is an increase in transcription of the *tcdA* and *tcdB* genes [89], suggesting that the toxin may play a role in transmission by disrupting the intestinal epithelial layer once bacterial propagation slows. Diarrhea from the subsequent accumulation of fluid in the intestine then releases the pathogen back into the environment. Within this life cycle, TcdB is recognized as a key component contributing to bacterial proliferation and has therefore become an important target for the treatment of *C. difficile* infections [84]. Neutralizing antibodies have been shown to inhibit TcdB toxicity; however, strain-specific variants of the toxin may not share identical neutralizing epitopes [90], necessitating further exploration of potential inhibitors.

## Disruption of membrane-specific interactions as a means of inhibiting toxin activity

While each of the organisms described above produce multiple virulence factors, the toxins described here play a key role in bacterial colonization, survival within the host, and/or transmission to another host. For this reason, inhibition of the activity of these toxins represents a means of limiting pathogen colonization and/or transmission to prevent, treat, or limit the severity of disease.

In their initial interaction with host cells, each of these bacterial toxins must recognize specific lipid and/or protein component(s) on the host cell membrane to initiate the often complex mechanism of host cell intoxication. These steps have often been well-studied, enabling the identification of putative therapeutic targets to inhibit toxin interactions with host cells. A number of strategies have been investigated, depending on the exact mechanism of each toxin, as described below.

### Receptor-based molecules

One effective strategy for inhibiting toxin activity is to employ a toxin’s affinity for a specific target against it. Receptor-based molecular inhibitors are purposely designed to mimic the target to compete for toxin binding. Essentially, this strategy introduces “decoy” receptors that render the toxin inert by binding to the receptor binding site, thus halting the cytotoxic activity.

#### Cholera toxin

**Fig. 1:**
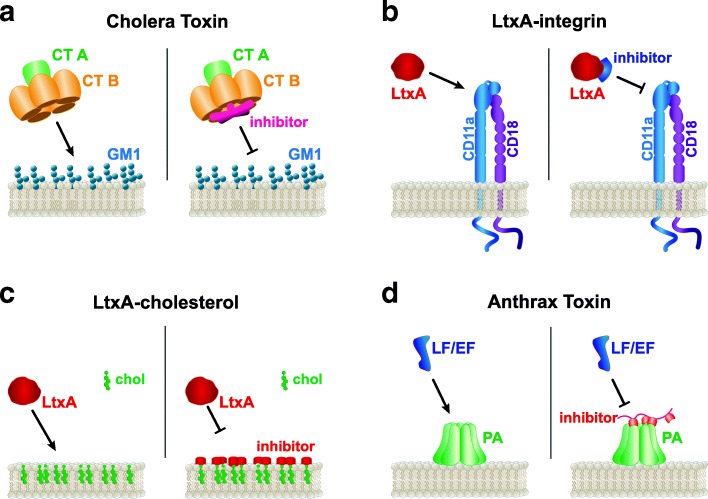
Receptor-based inhibitors. **a** An engineered multivalent ligand inhibits the CT B subunit from interacting with GM1 on the host cell membrane. **b** A small peptide based on the binding site of the integrin CD11a targeted by LtxA inhibits toxin binding to the receptor. **c** A peptide inhibitor based on the CRAC motif of LtxA shields cholesterol in the host membrane. **d** A polyvalent inhibitor blocks LF and EF from interacting with membrane-bound PA

The B pentamer of CT targets the pentasaccharide headgroup of the ganglioside GM1, leading to investigations into the specific structural elements that contribute to the binding. Based on this, Minke and colleagues concluded that galactose derivatives, such as m-nitrophenyl-α-D-galactoside, presented an encouraging template on which to base a CT inhibitor [91]. In subsequent studies, this group engineered branched multivalent ligands to match the 5-fold symmetry of the CT B pentamer binding sites (Fig. 1a). They showed that synthesis of pentavalent and decavalent ligand structures were able to form 1:1 and 1:2 ligand:toxin complexes, respectively, and achieve affinities on the order of the affinity of the CT B pentamer with GM1 [92]. Interestingly, they found that a similar strategy could be used to inhibit the heat-labile enterotoxin (LT) from *Escherichia coli* because the initial binding mechanism of this toxin is very similar to that of CT [93, 94]. Other toxins, such as Shiga and pertussis toxins [95], share this AB5 structure, opening this type of geometry-based inhibitor to broader applications using a similar design approach.

#### *Aggregatibacter actinomycetemcomitans* leukotoxin

The white blood cell specificity of LtxA derives from its recognition of the LFA-1 integrin, which is only expressed by human white blood cells. This integrin is a heterodimer composed of CD11a (αL) and CD18 (β2) subunits [96, 97]. Investigations into the specific binding domain of LtxA on LFA-1 used a series of transfection experiments to narrow the location to the first 128 amino acids on the N-terminal of the CD11a subunit [38]. The authors further speculated that the domain should reside between residues 58–128 due to the location of an epitope and the inability of that monoclonal antibody to inhibit LtxA-mediated cytotoxicity. This location suggests that the binding site is situated on β-sheets 1 and 2 of the β-propeller domain of the CD11a subunit [38, 98, 99]. Following this structural information, our group synthesized peptides corresponding to the individual β-strands in β-sheets 1 and 2 and demonstrated their ability to inhibit LtxA-mediated cytotoxicity (Fig. 1b) [100]. The affinity of LtxA for each of the peptides was determined to drive the inhibitory activity of the receptor-based molecules [100].

In addition to the interaction with LFA-1, LtxA also requires the presence of cholesterol in the host cell plasma membrane [34]. The recognition of cholesterol by the toxin is mediated by a cholesterol recognition amino acid consensus (CRAC) domain within the primary structure of protein. Using this domain as the template for a synthetic peptide, we designed a cholesterol-binding peptide to block the recognition of cholesterol by LtxA. When this peptide was incubated with leukocytes, the cytotoxicity of LtxA was completely inhibited by blocking its interaction with cholesterol [101, 102]. Essentially, the CRAC peptide competes with the toxin for cholesterol in the cell membrane “using up” all of the potential binding sites (Fig. 1c). Importantly, cells treated with the CRAC peptide did not have a significant difference in viability over 65 days compared to an untreated control, suggesting this treatment has minimal long term effect on host cells [102].

#### Anthrax toxin

In the initial interaction of the tripartite anthrax toxin with host cells, the PA domain must recognize either ATR/TEM8 or CMG2 on the host cell [47, 48], and upon subsequent oligomerization, the enzymatic LF or EF domains can bind [103]. This mechanism thus provides several inhibitory possibilities, including inhibition of the initial interaction of PA with its receptor(s). Toward this end, soluble proteins containing the putative PA binding domains of both ATR/TEM8 and CMG2 (sATR/TEM8 and sCMG2, respectively) were produced and compared in terms of their abilities to inhibit EF/LF binding and intoxication. The sCMG2 protein was found to bind more strongly to PA than the sATR/TEM8 protein and as a result, was more effective in preventing EF-mediated cytotoxicity. This protein was also effective in preventing anthrax toxin-mediated death in an in vivo rat model [104]. A subsequent study found that this inhibitor was also effective against four engineered, antibody-resistant forms of PA [105], demonstrating the utility of a receptor-based inhibitor approach in cases where neutralizing antibodies are ineffective.

In an alternative strategy, a polyvalent inhibitor has been shown to successfully inhibit the action of the anthrax toxin through interaction with the PA heptamer, preventing assembly of the final complex [106]. In this study, the researchers identified peptides that bound exclusively to the PA heptamer at or near to the EF/LF binding site. They then synthesized a polyvalent inhibitor by linking copies of the peptide to a polyacrylamide molecule and showed that the molecule can prevent LF binding to the PA heptamer, resulting in the inhibition of cytotoxicity (Fig. 1d) [106].

### Assembly inhibitors - dominant-negative inhibitors

Many toxins, as part of the cytotoxic activity, require assembly or oligomerization. An interesting strategy to target toxins that utilize this pathway during their interaction with a host is through altering toxin subunits with point mutations at strategic domains. Although there may be many mutations that render a toxin inactive, a mutant dominant-negative toxin must still interact with the wild-type (WT) toxin and may still interact with the host cells. The combination of the dominant-negative toxin and the WT then assemble into an inactive hybrid toxin complex, inhibiting the activity of the WT toxin.

#### *Staphylococcus aureus* leukocidins

**Fig. 2:**
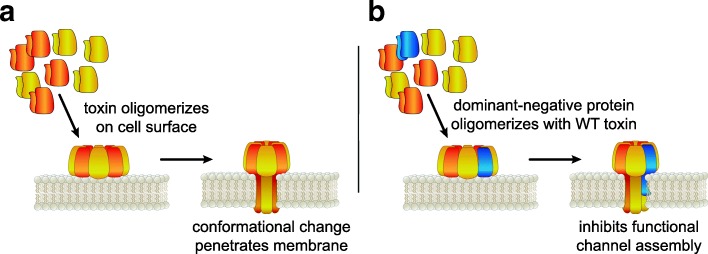
Dominant-negative inhibitors. **a** WT toxin in solution forms an oligomer on the host cell surface. After a conformational change, the transmembrane domains assemble a channel in the plasma membrane. **b** Incorporation of a dominant-negative protein with WT toxin prevents cytotoxic activity by inhibiting the assembly of a functional channel

The subunit monomers of the *S. aureus* S- and F-class leukocidins contain a glycine-rich motif localized in what will assemble into the stem domain of the β-barrel pore (Fig. 2a). Investigations by Reyes-Robles and colleagues revealed that these motifs are critical for toxin activity, demonstrating that cells incubated with 5–6 residue deletion mutations of the S- and F-class subunits were not killed [51]. They also showed that mixing the mutant subunits with the WT toxin prevented cell lysis, suggesting that the mutants exhibited a dominant-negative effect by neutralizing the WT toxin. Their analysis on the mechanism of this inhibition suggests that the dominant-negative mutant and WT subunits continue to oligomerize but assemble into a defective pore complex, thus inhibiting toxicity (Fig. 2b) [51].

#### *Clostridium perfringens* ε-toxin

Similar to the *S. aureus* LukF toxin, the ε-toxin of *C. perfringens* contains a membrane-penetrating domain that unfolds after oligomerization on a host cell membrane and forms a channel. An investigation by Pelish and McClain targeted this conformational change to inhibit channel insertion by developing a mutated toxin containing strategically substituted cysteines at locations in the membrane insertion domain and in the protein backbone predicted to form disulfide bonds [107]. These mutations constrained the protein in its globular form, inhibiting toxin activity by preventing the conformational change required for membrane insertion. They report that the mutated protein had no cytotoxic activity, and more importantly, when incubated with WT toxin, exhibited a dose-dependent inhibition of cytotoxicity. They also determined that the WT toxin retained its ability to bind to the host cells in the presence of the mutant protein. Their further analysis suggests that the mechanism of inhibition is through the formation of mixed oligomeric complexes containing active WT and inactive mutant toxins. By focusing on restricting an alteration in the protein’s secondary structure, the authors demonstrated that a toxin’s targeted receptor may not need to be known or understood to develop a method of inhibiting toxin activity.

#### *Helicobacter pylori* VacA

An investigation into the role of a hydrophobic region near VacA N-terminus found that a deletion mutant (VacA-(Δ6–27)) of the toxin inhibited the activity of the WT. Vinion-Dubiel and colleagues reported that the secretion and oligomerization of VacA-(Δ6–27) was indiscernible from that of the WT. However, after internalization by cells, the mutant lacked vacuolation and cytotoxic activity [73]. Furthermore, they found that when mixed with WT toxin, VacA-(Δ6–27) also exhibited a dominant-negative effect, inhibiting the cytotoxic activity of the active toxin. Their results indicate that the deleted domain is important for the functional activity of the toxin. Subsequent investigation revealed that three GXXXG motifs, missing in the deletion mutant, are critical for the membrane channel assembly [108]. Similarly, Genisset and colleagues developed a VacA deletion mutant, instead focusing on a region known to be protected from proteolysis [109]. The secretion of this mutant, VacA Δ49–57 was also indiscernible from that of the WT toxin, but the mutant failed to oligomerize, resulting in an absence of cytotoxic activity. The authors reported that VacA Δ49–57 was internalized by the cells similarly to that of the WT toxin, but did not form oligomeric structures. They also demonstrated that the mutant toxin was able to prevent the cytotoxic activity of the WT toxin in a concentration-dependent manner, suggesting that VacA Δ49–57 exhibits a dominant negative effect.

### Membrane-based decoys

**Fig. 3:**
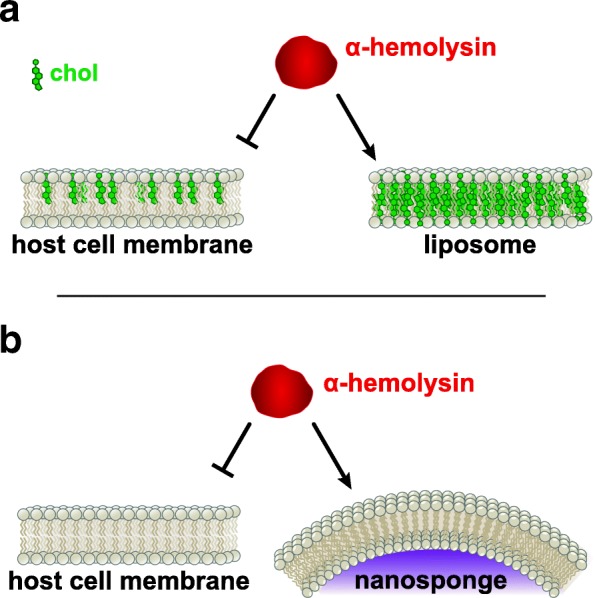
Membrane-based inhibitors. **a** Many toxins, including *S. aureus* α-hemolysin, bind preferentially to cholesterol-containing membranes. A liposome with an unnaturally high cholesterol composition was demonstrated to absorb α-hemolysin, preventing its interaction with host cells. **b** A nanosponge was created in which a red blood cell membrane was fused to a PLGA nanoparticle core. This particle was more effective in inhibiting α-hemolysin from interacting with host cells than either liposomes or red blood cell membrane vesicles not fused to the polymer core

Many toxins have been demonstrated to interact specifically with the cholesterol- and sphingolipid-rich regions of the plasma membrane known as lipid rafts. To take advantage of this, Henry et al. developed a liposome composed of 66% cholesterol and 34% sphingomyelin, the maximal cholesterol composition of a liposome [110], to sequester multiple cholesterol-binding toxins, including the *S. aureus* α-hemolysin, several CDCs (streptolysin O, tetanolysin, pneumolysin) and phospholipase C. When these toxins were incubated in a culture containing both liposomes and THP-1 cells, the toxins bound primarily to the liposomes, leaving the cells unaffected (Fig. 3a). The authors found that the particularly high cholesterol composition was required for the inhibitory activity, as liposomes without cholesterol were either ineffective or had limited protective effect on the various toxins. In a series of co-culture experiments, the cholesterol/sphingomyelin liposomes provided complete protection of THP-1 cells from the toxins secreted by *Streptococcus pyogenes*, but protection of the cells from the toxins secreted by methicillin-resistant *S. aureus* (MRSA) required both the cholesterol/sphingomyelin liposomes along with sphingomyelin-only liposomes, suggesting that this organism may secrete two different toxins, with different membrane affinities. A combination of cholesterol/sphingomyelin and sphingomyelin liposomes was also effective in protecting cells against *S. pneumoniae* as well as clinical *S. aureus* strains. This liposome combination was likewise effective in multiple in vivo models of disease, including an invasive pneumococcal pneumonia model and a fatal pneumococcal sepsis model. The authors additionally demonstrated that low doses of their liposomal mixture, along with a low dose of antibiotic, was able to treat sepsis caused by *S. pneumoniae* in a mouse model [111].

Hu et al. adopted a related strategy to treat *S. aureus* infections by taking advantage of the affinity of the α-hemolysin for plasma membrane lipids. In this group’s approach, which they called a “nanosponge,” a red blood cell membrane was fused to a poly(lactic-*co*-glycolic acid) (PLGA) nanoparticle core (Fig. 3b). The nanosponge protected red blood cells from α-hemolysin-mediated lysis, while uncoated PLGA nanoparticles, liposomes, and red blood cell vesicles were unable to protect the cells. While both the red blood cell vesicles and nanosponges were able to absorb the toxin, only the nanosponges retained the toxin, preventing its subsequent interaction with host cells. The efficacy of the nanosponges was also demonstrated in two in vivo α-hemolysin models [112].

Polymers have also been used to absorb toxins, thus preventing their interactions with host cells. Tolevamer is an anionic polymer of high molecular weight produced by Genzyme. This polymer strongly binds both the A and B toxins produced by *C. difficile* [113] and as a result, inhibits the activity of the toxin against host cells [114]. This group demonstrated that the polymer is effective in reducing the toxicity of a *C. difficile* infection in a hamster model [114]. This behavior appears to be unique to this particular polymer, as another anionic polymer, poly(2-acrylamido-2-methyl-1-propanesulfonate) (AMPS), did not have the same effect [113].

### Inhibiting membrane-perforating toxins

Many of the cytotoxic pathways involve the insertion of the toxin into the plasma membranes of the host cells, forming channels for the translocation of enzymatic toxin domains or the disruption of the cellular electrochemical potential. A technique that has also proven useful for halting this cytotoxic pathway is to physically block the channel. In some ways, this technique is already in common usage across different living systems; many organisms make toxins that specifically target channels to inhibit cellular function, leading to paralysis, organ failure or death [115–117]. Using an analogous approach to interrupt one of the major steps in the cytotoxic pathways of pore-forming toxins, targeting the channel conductivity has been demonstrated as an effective anti-virulence strategy. The advantage of this anti-toxin approach is that it can be used to treat established infections after the toxins have already been released, a limitation of most other strategies, which are most effective when used prophylactically.

#### Anthrax PA channels

**Fig. 4:**
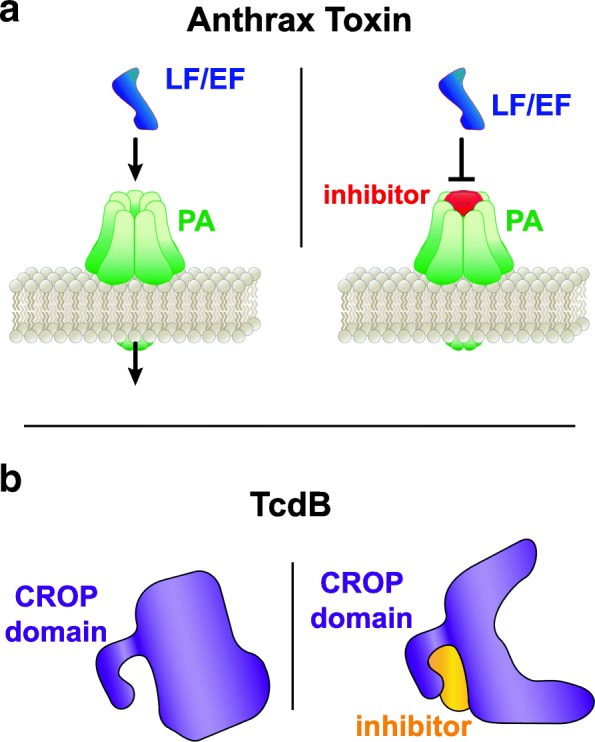
Inhibition of pore formation and requisite conformational changes. **a** Blocking the pore formed from the anthrax PA toxin inhibits translocation of the enzymatic subunits (EF and LF) into the host cell. **b** A peptide inhibitor binds to the CROP domain of the TcdB toxin, destabilizing the protein by preventing the conformational changes required for cytotoxic activity

As discussed earlier, the PA toxin from *B. anthracis* is required for the translocation and cytotoxicity of the enzymatic ET and LT toxins. The pore formed by the PA assembly contains negatively charged domains and exhibits a heptameric symmetry. In trying to inhibit the activity of LT by preventing its entry into the cell, Moayeri and colleagues used this structural information to develop a β-cyclodextrin derivative to block the PA pore (Fig. 4a) [118, 119]. They found that rats treated with the antitoxin lived significantly longer than those treated with PBS [118]. They also reported that the combination of antitoxin and the antibiotic ciprofloxacin given one day after *B. anthracis* infection significantly protected mice against the infection compared to ciprofloxacin alone [118]. Antibiotic treatments have very low effectiveness against *B. anthracis* infection once symptoms begin due to the concentration of toxin already produced [119]. Therapies like this, which incorporate antitoxin strategies to inhibit transport of the enzymatic toxin domains, fulfill an important deficiency in the treatment of *B. anthracis* infection.

#### *Clostridium perfringens* ε-toxin

There are currently no available treatments that are effective after infection with the *C. perfringens* ε-toxin. The toxin oligomerizes and forms large pores in the plasma membrane of host cells, disrupting the electrochemical gradients of the cells and leading to cell death. *C. perfringens* infection is common in livestock and the current treatments are entirely prophylactic, as vaccines and antitoxin sera do not protect the animal after ε-toxin infection symptoms appear, suggesting they are not effective once the toxin has formed a channel [120]. Since there are limited veterinary treatment options and none for human use, the development of an antitoxin therapy would be a significant advancement in treating this powerful toxin. Lewis and colleagues screened over 150,000 potential small molecules for their ability to block ε-toxin channels. They reported two compounds, 4-*tert*-butyl-N-cyclooctylbenzamide and *N*-methylfuro[2,3-*b*]quinolone-2-carboxamide, compounds I and II respectively, which were able to inhibit transport through ε-toxin channels [120]. They then tested 43 additional analogues of compound I to identify a relationship between structure and inhibition activity, identifying seven compounds that could inhibit cytotoxicity. Their result also suggests that none of the compounds interfered with the toxin’s ability to bind to the cell or form a pore, and the authors therefore hypothesized that the compounds interfere with the pore itself.

### Targeting toxin conformation

Differences in TcdB activity between hypervirulent and historically non-epidemic strains of *C. difficile* have been associated with structural variations in the CROP domains, resulting in differences in the pH required for cell entry [121]. Lanis and colleagues found that sequence differences in TcdB from the hypervirulent strain (TcdB_HV_) permitted conformational changes at higher pH, resulting in an earlier translocation of the toxin into the cytosol during endocytosis. Additionally, further work from the same group identified that conformational variations observed in TcdB_HV_ can also conceal epitopes from neutralizing antibodies known to target historically non-epidemic strains [122]. Their studies revealed that differences in the sequence of the TcdB_HV_ permitted stronger intramolecular bonding, giving rise to a conformation that shields the neutralizing epitopes. With this information, the researchers engineered a series of peptides based on the toxin’s structure to interrupt the intramolecular interactions and destabilize the toxin to inhibit activity (Fig. 4b) [123]. They identified an 11-amino acid consensus sequence within several peptides that were able to protect cells from TcdB and reported that one peptide in particular formed multiple interactions with the toxin. Interestingly, they did not find that the peptide had any effect of the enzymatic activity of the toxin, but did identify that the peptide-mediated destabilization made the toxin more susceptible to proteolysis.

### Small molecules to alter toxin conformation and activity

**Fig. 5:**
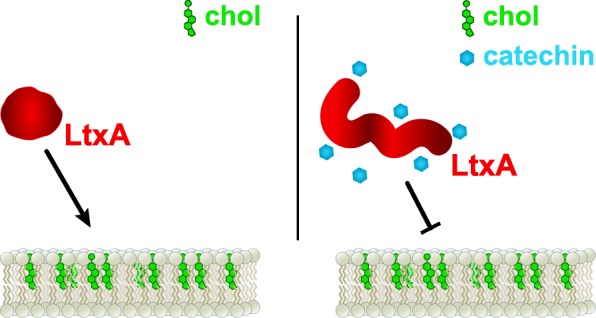
Natural-product mediated conformational changes. EGCg induces significant conformational changes in LtxA, resulting in a substantial decrease in the ability of the toxin to bind cholesterol in the host cell plasma membrane, and as a result, inhibiting activity of the toxin

(−)-Epigallocatechin gallate **(**EGCg), a polyphenol found in tea, was found to inhibit the intracellular survival of *Listeria monocytogenes* within macrophages. This organism secretes a toxin, listeriolysin O (LLO), a member of the cholesterol-dependent cytolysin family, which facilitates the escape of the bacterium from the phagosome, allowing the bacterium to reach the cytoplasm [124]. The researchers discovered that EGCg disrupted the ability of LLO to bind membrane cholesterol, thus preventing phagosome disruption and bacterial survival [125]. We have recently discovered a similar effect of EGCg on LtxA produced by *A. actinomycetemcomitans*. Like LLO, LtxA requires recognition of host cell plasma membrane cholesterol in order to intoxicate the cell. EGCg significantly altered the conformation of LtxA, resulting in reduction of cholesterol binding and subsequent toxin-mediated cytotoxicity (Fig. 5) [126]. Similarly, grape extract, which includes EGCg, among other molecules, was found to inhibit the activity of a number of toxins, including Shiga toxin [127], LT, and CT [128]. The mechanism of inhibition appears to be consistent with that observed with LtxA; the extract alters the conformation of CT, preventing the toxin’s binding to its receptor, GM1 [128, 129]. Likewise, EGCg and other polyphenolic molecules were found to inhibit the *H. pylori* VacA toxin [130].

A similar naturally occurring polyphenol, apigenin, has been shown to inhibit the cytotoxicity of Ply from *S. pneumoniae*. Song and colleagues demonstrated that apigenin inhibited the oligomerization of Ply, neutralizing its lytic activity on human lung epithelial cells in vitro by preventing the assembly of functional pores [131]. When investigated in vivo, they discovered that mice that received subcutaneous injections of apigenin had a significantly lower bacterial burden after 48 h than control mice following intranasal infection with *S. pneumoniae*. They also found significantly lower levels of the cytokines tumor necrosis factor α (TNF-α) and interleukin 1β (IL-1β) in the bronchoalveolar lavage fluid from the apigenin-treated mice, suggesting that they experienced less bronchial inflammation. A separate study investigating the effects of apigenin in vitro and in vivo made similar conclusions regarding α-hemolysin from *S. aureus*. This group reported that subcutaneous injections of apigenin significantly reduced the bacterial burden from intranasal infection with *S. aureus* [132]. However, unlike the anti-oligomerization mechanism for Ply from *S. pneumoniae* infection, they attributed the mechanism of apigenin on *S. aureus* to the decreased α-hemolysin production. Together, these results demonstrate that small molecules, and more specifically naturally occurring compounds, possess significant potential for combating bacterial infection.

## Conclusions and future perspectives

With the current rise in antibiotic resistance, new approaches to treat diseases caused by bacteria are urgently needed. Anti-virulence strategies present a promising approach to this issue, by eliminating the advantages provided by certain virulence factors to pathogenic bacteria, thus promoting natural clearance mechanisms. As part of their pathogenic mechanism, a number of bacteria secrete toxins to interact directly with host cells. To initiate the process of cell intoxication, each of these toxins must recognize at least one specific element on the host cell, using mechanisms that have been well studied over the years, leading to the identification of possible therapeutic targets.

We have described here multiple strategies to utilize these known mechanisms to create specific inhibitors of bacterial toxins by interfering with the recognition of host cell membrane components by the toxin. Success has been demonstrated in vitro and often in vivo against a range of toxins and bacteria. However, none have been approved for clinical use, a fact that could be due to both design and testing concerns that must be addressed in order for the next phase of inhibitors to find clinical success.

Pathogenic bacteria often produce an arsenal of virulence factors, and sometimes even multiple toxins. Thus an anti-toxin strategy can only be effective if the targeted toxin plays a key role in the pathogenicity of the organism. In cases where the bacterium secretes multiple toxins, each playing important roles in pathogenesis, multiple inhibitors used in combination may be required. Additionally, it has recently been established that some of these toxins are secreted in the well-studied free form, as well as in association with membrane vesicles. Our lab has recently demonstrated that in their vesicle-associated form, CT and LtxA interact with host cells in a manner that does not require the receptor of the free toxin (GM1 and LFA-1/cholesterol, respectively) [133, 134]. In other words, a single bacterium can release the same toxin in multiple forms, each with distinct pathways of internalization; multiple inhibitors may therefore be necessary to inhibit even a single toxin.

Additional complicating factors relate to the necessary concentrations of these inhibitors. The expression of virulence factors, including toxins, is regulated by environmental conditions and will therefore vary throughout the course of an infection. It is difficult to know what the in vivo toxin concentration will be, thus complicating the determination of minimum inhibitory concentrations. As with all therapeutic entities, toxic and/or off-target effects are possible and must be considered during the design and testing of these inhibitors. Strategies that target an element on the toxin itself rather than the receptor on the host cell are preferable, for this reason.

In addition to these therapeutic design issues, there exist difficulties in the design of clinical trials due to two primary issues. First, because these molecules do not directly mediate bacterial death, we do not currently have well-defined metrics of success for the drugs. For example, rather than measuring decreased bacterial burden upon treatment, an appropriate metric might be a reduction in disease severity or an enhanced immune response. Currently, these metrics are not well quantifiable. Additionally, the response of the bacteria to the drugs is likely slower than the response to traditional antibiotics. Thus, before large-scale trials can be initiated, the field must first define those factors that indicate successful treatment. Second, most of these anti-toxin and anti-virulence strategies have been developed in academic labs, which generally lack the funds to complete large-scale clinical trials. Therefore, to demonstrate the usefulness of these new molecules in human patients, industrial collaborations will be essential.

Despite these limitations and complications, the promise of anti-toxin strategies is great, as these molecules provide specific, targeted activity and are less likely to lead to the negative side effects associated with traditional antibiotics, which are often caused by nonspecific killing of bacterial cells. These approaches would spare the host microbiota, affecting only the pathogenic bacteria. Additionally, because the molecules do not directly kill the targeted bacteria, the selective pressure is reduced compared to traditional antibiotics, thus limiting the rate at which resistance will develop. One particularly promising approach that has been demonstrated is the use of anti-toxin strategies in combination with more traditional antibiotics to reduce the concentration of antibiotics needed to clear an infection. We anticipate that because of these benefits, with additional focused study, anti-toxin molecules will soon reach clinical use with great impact on the treatment of infectious disease.

## Abbreviations

AMPS: Poly(2-acrylamido-2-methyl-1-propanesulfonate)
ATR: Anthrax toxin receptor
CDC: Cholesterol-dependent cytolysin
CMG2: Capillary morphogesis protein 2
CRAC: Cholesterol recognition amino acid consensus motif
CROP: Combined repetitive oligopeptides
CT: Cholera toxin
EF: Edema factor
EGCg: (−)-epigallocatechin gallate
HlgAB/CB: γ-hemolysin (HlgAB/CB)
HPNAP: *H. pylori* neutrophil activating protein
IL-1β: Interleukin 1β
LF: Lethal factor
LFA-1: Lymphocyte function-associated antigen-1
LLO: Listeriolysin
LT: Heat-labile enterotoxin
LtxA: Leukotoxin
LukAB: LeukocidinAB
LukED: LeukocidinED
LukSF: Panton-Valentine leucocidin
MRSA: Methicillin-resistant *Staphylococcus aureus*
PA: Protective antigen
PLGA: Poly(lactic-*co*-glycolic acid)
Ply: Pneumolysin
TcdA/B: *Clostridium difficile* toxin A/B
TEM8: Tumor endothelial marker 8
TNF-α: Tumor necrosis factor α
VacA: Vacuolating toxin
WT: Wild-type
